# GADTI: Graph Autoencoder Approach for DTI Prediction From Heterogeneous Network

**DOI:** 10.3389/fgene.2021.650821

**Published:** 2021-04-09

**Authors:** Zhixian Liu, Qingfeng Chen, Wei Lan, Haiming Pan, Xinkun Hao, Shirui Pan

**Affiliations:** ^1^School of Medical, Guangxi University, Nanning, China; ^2^School of Electronics and Information Engineering, Beibu Gulf University, Qinzhou, China; ^3^School of Computer, Electronic and Information, Guangxi University, Nanning, China; ^4^Department of Data Science and AI, Monash University, Melbourne, VIC, Australia

**Keywords:** drug-target interaction prediction, network embedding, graph convolutional network, autoencoder, random walk, heterogeneous network

## Abstract

Identifying drug–target interaction (DTI) is the basis for drug development. However, the method of using biochemical experiments to discover drug-target interactions has low coverage and high costs. Many computational methods have been developed to predict potential drug-target interactions based on known drug-target interactions, but the accuracy of these methods still needs to be improved. In this article, a graph autoencoder approach for DTI prediction (GADTI) was proposed to discover potential interactions between drugs and targets using a heterogeneous network, which integrates diverse drug-related and target-related datasets. Its encoder consists of two components: a graph convolutional network (GCN) and a random walk with restart (RWR). And the decoder is DistMult, a matrix factorization model, using embedding vectors from encoder to discover potential DTIs. The combination of GCN and RWR can provide nodes with more information through a larger neighborhood, and it can also avoid over-smoothing and computational complexity caused by multi-layer message passing. Based on the 10-fold cross-validation, we conduct three experiments in different scenarios. The results show that GADTI is superior to the baseline methods in both the area under the receiver operator characteristic curve and the area under the precision–recall curve. In addition, based on the latest Drugbank dataset (V5.1.8), the case study shows that 54.8% of new approved DTIs are predicted by GADTI.

## Introduction

The drug acts on the target protein, thereby affecting the expression of the target protein to achieve the therapeutic effect on the disease. Therefore, finding drug-target interactions is the basis of drug development. The research and development of innovative drugs often requires billions of dollars and more than a decade of work, and usually ends in failure. Hence, it is an important choice for pharmaceutical companies to discover potential drug–target interactions (DTIs) by using the known DTIs. The properties of existing drugs are familiar to people, and their safety is guaranteed. However, there are some limits in both coverage and throughput of biochemical experiments to identify new DTIs. Consequently, the prediction of DTIs using computational methods has attracted extensive attention.

Early computational methods were mainly based on drug–drug similarity and target–target similarity, or the features of drugs and targets. Some of the methods based on similarity first calculate the similarity between the drug pairs (e.g., chemical structure similarity) and the similarity between the target pairs (e.g., protein sequence similarity), and then use the known DTIs to score the unknown DTI (Cheng et al., 2012; Mei et al., 2013; Wang et al., 2014). Other similarity-based methods process a random walk on the network composed of multiple data sources, such as drug–drug interactions, target–target interactions, and DTIs to obtain the similarity between nodes to predict new DTIs (Chen et al., 2012; Seal et al., 2015). In the methods based on features, both the drugs and the targets are represented as fixed-length feature vectors, and the known drug–target pairs are divided into positive and negative categories. Then the DTI prediction is transformed into a binary classification problem. Machine learning methods such as support vector machines, random forests, and conditional random fields can be directly used for prediction (Nagamine et al., 2009; Lan et al., 2016; Olayan et al., 2018; Chen et al., 2019; Shi et al., 2019).

In recent years, network embedding methods (Perozzi et al., 2014) have shown excellent performance in network data analysis (Cai et al., 2017), and have been introduced into DTI prediction (Su et al., 2018; Bagherian et al., 2020; Liu et al., 2020). Network embedding is also known as graph embedding. In network embedding, nodes such as drugs and targets can all be converted into low-dimensional vectors that represent their features and can be directly used for DTI prediction. The main methods of network embedding include matrix factorization, random walk, and deep learning.

A multiple similarities collaborative matrix factorization model (Zheng et al., 2013) was proposed to predict DTI. It incorporates anatomical therapeutic chemical similarity and chemical structure similarity of drugs, as well as genomic sequence similarity, gene ontology (GO) similarity, and protein–protein interaction (PPI) network similarity of targets. A combination of these similarity matrices was used to approximate the drug feature matrix D and the target feature matrix T, and then the inner product between D and T was utilized to approximate the DTI matrix. TriModel (Mohamed et al., 2019) uses the drug-related knowledge graph to find potential DTIs. It learns the feature vectors of nodes in the knowledge graph through tensor decomposition. These vectors are used to determine whether the drug and the target interact. Meanwhile, DTINet (Luo et al., 2017) first uses the random walk to obtain the low-dimensional feature vector of each drug and protein, projects the drug vector and protein vector into the same space, and then discovers new interactions through matrix completion. Encouraged by the DeepWalk (Perozzi et al., 2014) model, some researchers have combined the random walk with shallow neural networks (Zong et al., 2017, 2019; Zhu et al., 2018). These methods first construct a heterogeneous network based on multiple data sources, and then apply DeepWalk, node2vec (Grover and Leskovec, 2016), and other algorithms to the network to obtain the embedding vectors of drug nodes and target nodes. NeoDTI (Wan et al., 2019) uses a deep learning method based on neighborhood information aggregation to discover new DTIs. It aggregates neighbor information based on edge types in heterogeneous networks. Then, the feature vector of the node is used to reconstruct the original network. There are also several studies based on drug structure and protein sequence (Wen et al., 2017; Karimi et al., 2019; Öztürk et al., 2019). Starting from the chemical structure and protein sequence of compounds, deep learning methods are then employed to predict drug–target binding affinity.

Matrix factorization methods can capture the global structure of the network, but its space complexity increases rapidly as the network scale increases. Random walk methods are more efficient because they usually gather only local features. Deep learning methods are outstanding in DTI prediction because it can discover hidden features and associations from multi-source heterogeneous network, and it is easy to integrate externally associated data of drugs and targets (e.g., GO) to improve performance. However, deep learning is computationally expensive and time-consuming. Among the deep learning methods, the graph convolutional network (GCN)-based message passing (also known as neighborhood information aggregation) algorithms have recently attracted special attention due to their flexibility and good performance (Kearnes et al., 2016; Ying et al., 2018; Wan et al., 2019). The GCN algorithms usually only consider the neighborhood with a short distance (e.g., the first-order neighborhood) because large distances will lead to over-smoothing, which degrades performance and increases computational complexity. However, the short distance easily leads to insufficient information about the neighborhood of the node (Li et al., 2018; Xu et al., 2018).

In this article, we propose a graph autoencoder approach for DTI prediction using a heterogeneous network (GADTI), which combines a graph convolutional network, matrix factorization, and random walk. GADTI first constructs a heterogeneous network that integrates eight data sources related to drugs and targets. Then, it runs a graph autoencoder model on the network to discover new DTIs. The encoder of the graph autoencoder includes two components: a GCN and a random walk with restart (RWR). The GCN component aggregates the first-order neighborhood information of each node and uses it to subsequently update the feature vector of nodes. The RWR component propagates the influence of nodes over the heterogeneous network. Through this, we obtain the embedding vectors of nodes, which are sent to the decoder. We use the matrix factorization model DistMult (Yang et al., 2015) to reconstruct the original heterogeneous network from the embedding vectors of nodes. Through the combination of GCN and RWR, GADTI can provide nodes with more information through a larger neighborhood while avoiding the over-smoothing and computational complexity caused by multi-layer message passing. The experimental results demonstrate that our approach is effective and efficient to predict potential DTIs.

## Materials and Methods

### Dataset

We adopted a dataset used in previous studies (Luo et al., 2017; Wan et al., 2019). It consists of eight networks, including four types of nodes (drugs, targets, diseases, and side effects) and eight types of edges (drug–drug interaction, DTI, drug–disease association, drug-side effects association, protein–protein interaction, protein–disease association, drug chemical structure similarity, and protein sequence similarity). These data come from public databases such as DrugBank, HPRD, and SIDER. The weights of edges in all networks are non-negative. Furthermore, only the drug chemical structure similarity and the protein sequence similarity are real-valued, and thus represent drug–drug chemical structure similarity scores and protein–protein sequence similarity scores. The others are binary values indicating whether there is an interaction or association between nodes. Table 1 lists the sources and statistics of these data.

**Table 1:** Sources of datasets and their statistical information.

**(a) Statistical information of nodes**		
**Node Type**	**Count**	
Drug	708	
Targets	1,512	
Disease	5,603	
Side effect	4,192	
**(b) Statistical information and source of edges**		
**Edge**	**Count**	**Data Source**
drug–target interaction	1,923	DrugBank v3.0 (Knox et al., 2011)
Drug–drug interaction	10,036	DrugBank v3.0 (Knox et al., 2011)
Protein–protein	7,363	HPRD Release 9 (Keshava Prasad et al., 2009)
Drug–disease	199,214	Comparative Toxicogenomics Database (Davis et al., 2013)
Drug side effect	80,164	SIDER Version 2 (Kuhn et al., 2010)
Protein–disease	1,596,745	Comparative Toxicogenomics Database (Davis et al., 2013)
Drug structure similarity	^*^	Based on Morgan fingerprints (Rogers and Hahn, 2010)
Protein sequence similarity	^*^	Based on Smith–Waterman scores (Smith and Waterman, 1981)
Total	1,895,445	

* *This edge is not counted because all node pairs are connected*.

### Spatial-Based Graph Convolutional Network

Most recent network embedding methods are based on the GCN, especially spatial-based GCN. These methods define convolution on graph as neighborhood information aggregation. They generate embeddings for nodes by aggregating the local neighborhood of the nodes instead of the entire network, which is regarded as a message passing mechanism.

A typical spatial-based GCN method includes two phases. In the initialization phase, it generates an initial vector based on the features of each node. If all the nodes in the network have no features, a one-hot vector is assigned to each node and a neural network is used to generate the initial vector. In the second phase, the vectors of nodes are updated by a combination of aggregated neighborhood vectors and the previous vectors of the nodes. These updates can be done through neural networks or linear transformations. The embedding vector of a node is a function of its neighborhood (including the node itself). This process looks similar to the receptive field of the convolution kernel in image processing, so it is called GCN. After one aggregation, the embedding vector of the node contains the feature information of its first-order neighbors. If we repeat this aggregation process *K* times, the embedding vector of the node can capture the feature information of its *K*-order neighbors. In the spatial-based GCN, the information of a node is first passed to its first-order neighbors, and then propagated to higher-order neighbors through edges on the network. Therefore, these methods are also called message passing methods. The process of graph convolution operation is summarized as follows:

(1) $$\begin{array}{l} a_{v}^{\left(n\right)}={\text{AGGREGATE}}^{\left(n\right)}\left(\left\{h_{u}^{\left(n-1\right)}:u\in N\left(v\right)\right\}\right),\;h_{v}^{\left(n\right)}\\ ={\text{UPDATE}}^{\left(n\right)}\left(\left\{h_{v}^{\left(n-1\right)},a_{v}^{\left(n\right)}\right\}\right) \end{array}$$

where AGGREGATE() and UPDATE ()are functions to aggregate neighborhood information and update node vectors, respectively; *u, v* are nodes; $a_{v}^{\left(n\right)}$ is the aggregated feature information of *v* at the *n*-th iteration; N(v) indicates the neighborhood of *v*; and $h_{v}^{\left(n\right)}$ is the embedding vector of *v* at the *n*-th iteration. After the iteration, we obtain $h_{v}^{\left(K\right)}$, which represents the features of *v* and can be directly used for node-level tasks such as node similarity calculations, node classification, and link prediction.

### Graph Autoencoder

The graph autoencoder takes the network and the feature vectors of the node as input to generate a low-dimensional embedding vector of the node or the entire network.

Unlike traditional autoencoders, the encoder of a graph autoencoder is usually a GCN and its variants, and the decoder can be an inner product (Kipf and Welling, 2016; Pan et al., 2018) or matrix factorization (Zitnik et al., 2018; Lan et al., 2020). Generative adversarial networks (GANs) (Goodfellow et al., 2014) and attention mechanisms have also been applied to graph autoencoders (Ma et al., 2018; Pan et al., 2018; Jin et al., 2019). For heterogeneous graphs containing multiple edge types, the encoder aggregates neighbor features one by one according to the edge type, and then merges them to obtain the embedding vectors of the nodes (Gligorijevic et al., 2018; Ma et al., 2018; Zitnik et al., 2018).

### GADTI

The data related to drugs and targets are represented in the form of a network, and the DTI prediction is then transformed into a link prediction of the network.

**Definition 1** Network *G* = (*V, R*), where *v* ∈ *V* and *r* ∈ *R* are nodes and edges, respectively.

**Figure 1:**
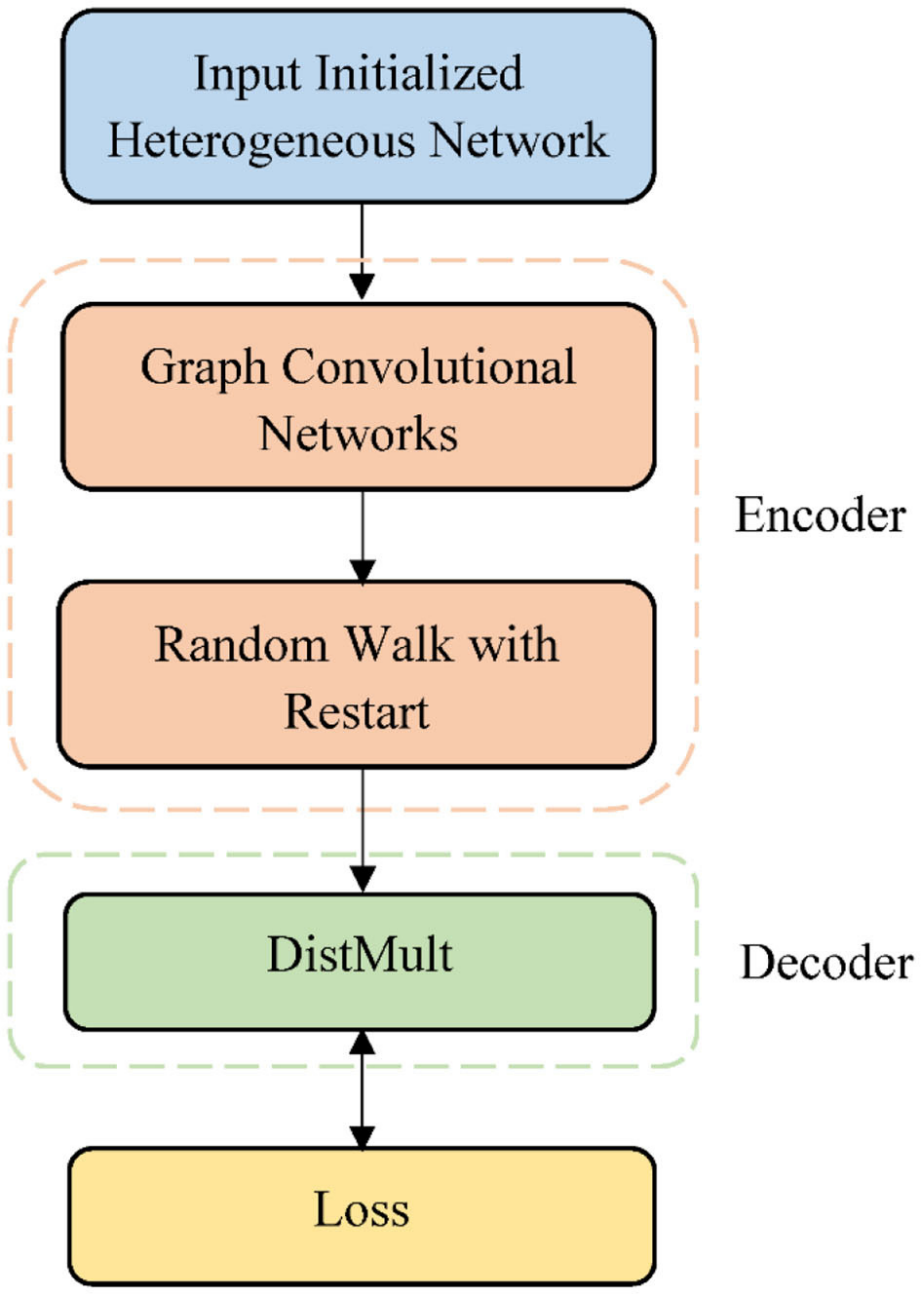
Overview of GADTI model architecture.

Given a network *G, v*_*d*_ and *v*_*t*_ are the drug node and target node, respectively. Our goal is to determine whether the unknown edge *r*_*dt*_ = (*v*_*d*_, *v*_*t*_) exists, or how likely it is to exist. To this end, we developed an end-to-end framework GADTI based on the graph autoencoder to discover new DTIs. This approach combines a graph convolutional network, matrix factorization, and random walk. GADTI first integrates multiple data sources to build a heterogeneous network, and then conducts prediction through a graph autoencoder model. As shown in Figure 1, GADTI has two main components:

- An encoder: a GCN followed by an RWR, which produces embeddings for nodes in *G*;
- A decoder: a matrix factorization model using these embeddings to predict DTIs.

### Encoder

The encoder consists of a GCN and an RWR. The GCN is used to aggregate first-order neighbor information to update node representation. Then, an RWR on the entire heterogeneous network allows the influence of nodes to spread far away so that we can obtain the final embedding vector. This approach can provide more information to nodes through a larger neighborhood while avoiding the over-smoothing and computational complexity caused by multi-layer convolutional networks.

#### Aggregation by GCN

In this stage, only the first-order neighborhood of the node is considered. For each node, we first group its first-order neighbors according to the type of edge. Then, for each neighbor group, a neighborhood aggregation operation is performed to aggregate information. Finally, the neighbor information of different groups is accumulated and concatenated with the previous embedding vector of the node, and then sent to the neural network to generate a new embedding vector. The process of aggregating and updating are defined as follows:

(2) $$\begin{array}{l} a_{v}^{r}=\sum_{u\in N_{r}(v)}\frac{1}{c_{r}^{v}}\sigma (W_{r}^{0}h_{u}^{0}+b_{r}),h_{v}^{*}\\ \;=MEAN\left(\left\{h_{v}^{0}\}\cup \{a_{v}^{r}:r\in R\right\}\right) \end{array}$$

where $a_{v}^{r}$ refers to the aggregated neighborhood information of *v* related to edge type *r*, $h_{v}^{0}\in {\mathbb{R}}^{d}$ refers to the initial embedding vector of *v*, *d* denotes the dimension of vector, *R* indicates the set of edge types, $N_{r}\left(v\right)$ are the neighbors of *v* related to edge type *r*, σ is a non-linear activation function, and $W_{r}^{0}\in {\mathbb{R}}^{d\times d}$ and $b_{r}\in {\mathbb{R}}^{d}$ are edge-type specific parameter matrix and bias terms used to aggregate neighborhood information, respectively. $c_{r}^{v}$ is a normalization constant that we choose to be $c_{r}^{v}=|N_{r}\left(v\right)|$. MEAN () is an element-wise mean operator, $h_{v}^{*}$ is the updated embedding vector of *v*.

**Figure 2:**
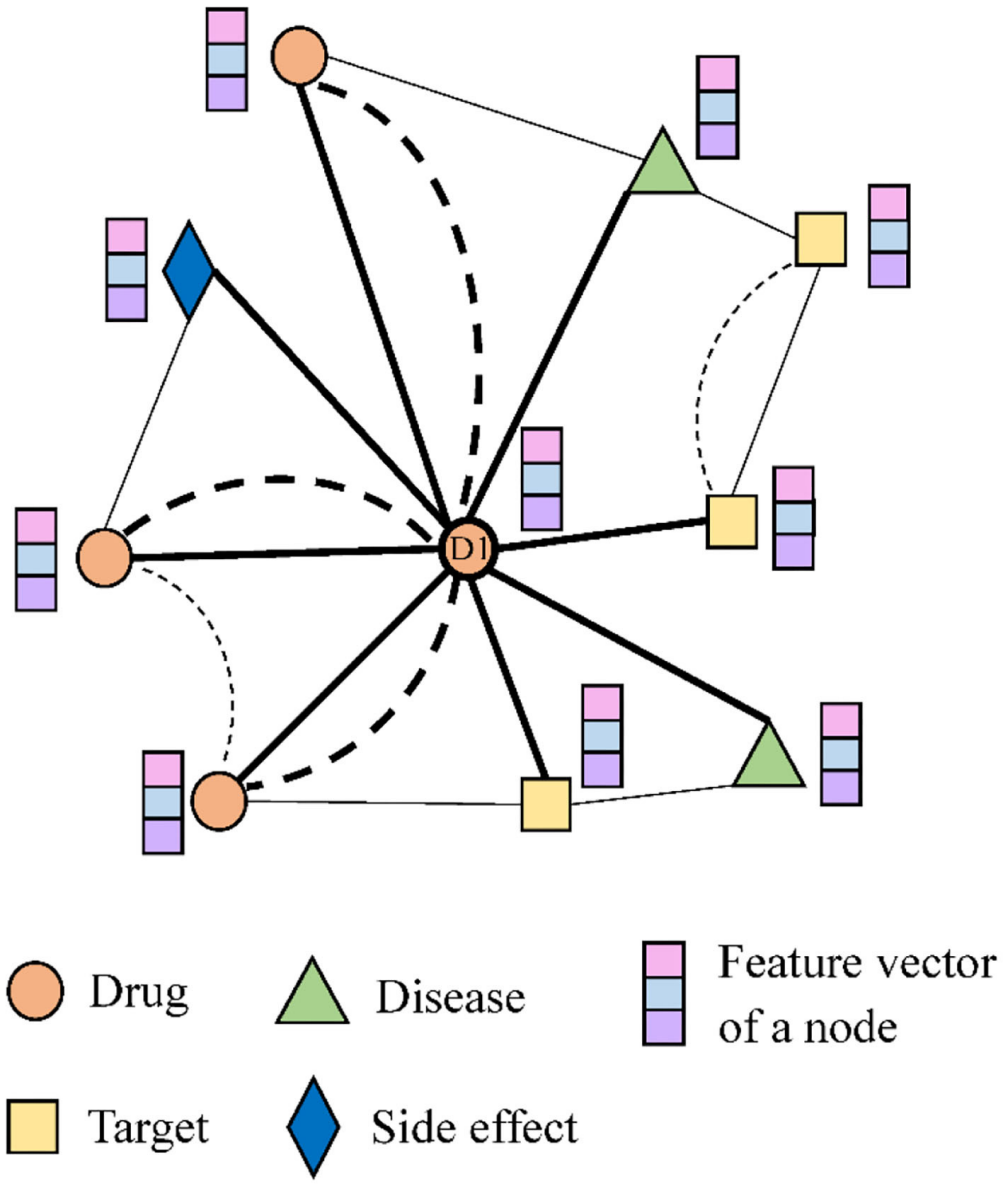
An small example of the heterogeneous network.

Figure 2 shows a small example of the network. Drug node *D1* is associated with two diseases and one side effect, as well as targets two proteins, and interacts with three other drugs. The bold dotted line indicates the similarity between drugs.

**Figure 3:**
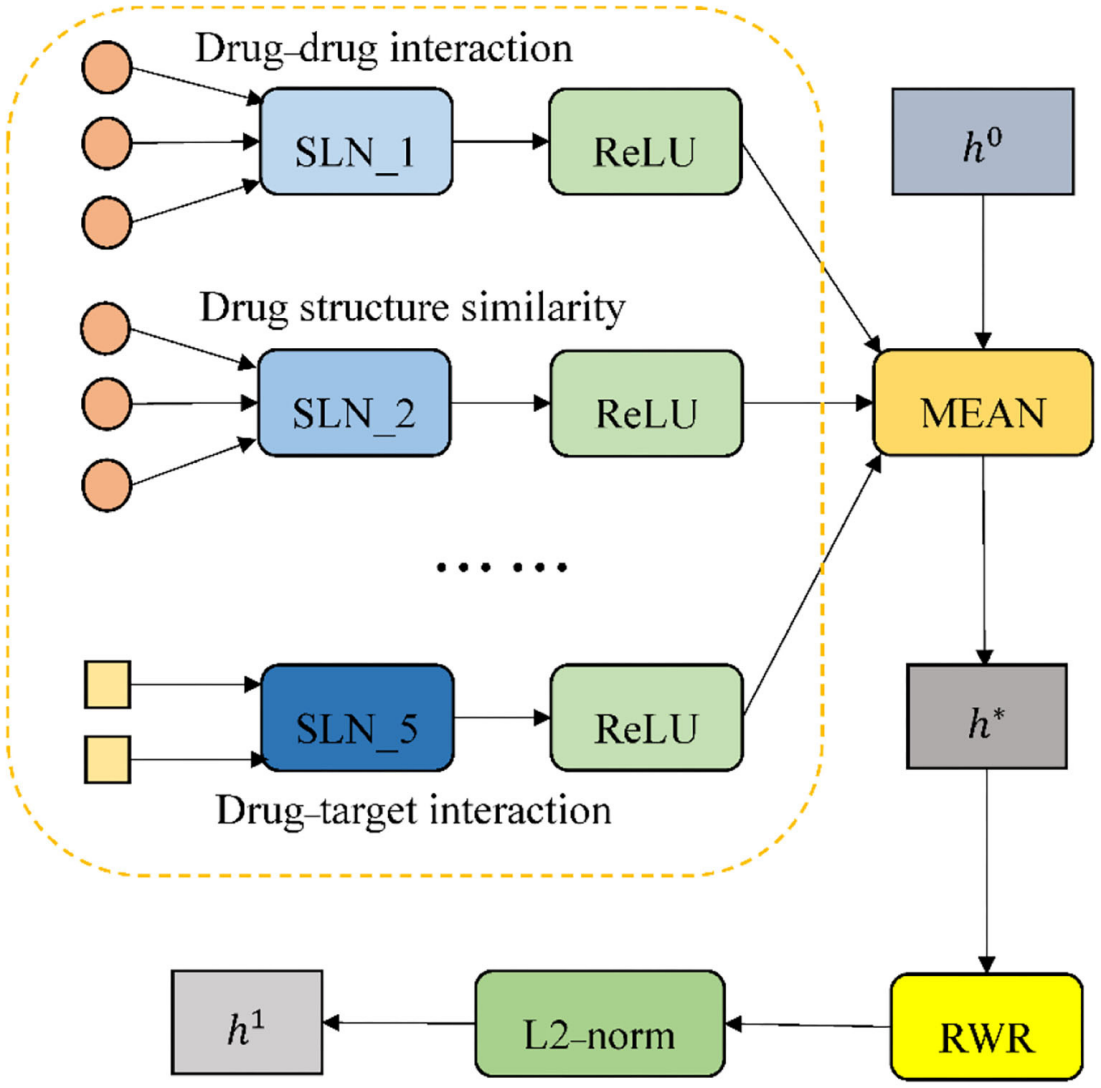
The process of the encoder (taking a drug node as an example).

The process of the encoder is provided in Figure 3. Multiple different single-layer neural networks (SLNs) are used in the encoder according to edge types. We take the drug node *D1* in Figure 2 as an example. Since there are five types of edges connected to *D1*, there will be five SLNs to aggregate neighbor information of corresponding edge types. The mean operator is chosen as the aggregation function, to perform an element-wise mean of the vectors in $\left\{h_{v}^{0}\}\cup \{a_{v}^{r}:r\in R\right\}$. It results in the new node embedding vector h^*^. *Relu* (*x*) = *max*(0, *x*) is selected as the element-wise activation function. A projection with learnable parameters is employed to initialize *h*^0^.

#### Propagation by RWR

The multi-hop neighborhood information aggregation implemented by multi-layer convolution often leads to over-smoothing. The aforementioned GCN only considers the one-hop graph structure, which causes the multi-hop information of the node to be underutilized. In order to solve this problem, we introduce an RWR, which spreads the influence of nodes to other nodes that are not directly adjacent through a walk on the heterogeneous network. The introduction of multi-hop information extends the range of information aggregation from the first-order neighborhood to the high-order neighborhood, which is equivalent to increasing the receptive field of convolution, thereby realizing long-range message passing.

Assuming that the transition matrix of the heterogeneous network is *A* and the restart probability is α, the RWR is defined as follows (Tong et al., 2008):

(3) $$\begin{array}{l} A_{ppr}=\alpha {\left(I-\left(1-\alpha \right)A\right)}^{-1} \end{array}$$

where *I* is the identity matrix, and *A*_*ppr*_(*u, v*) indicates the influence of node *u* on node *v*.

According to Equation (3), we can spread node information over long distances to get the final node embedding vector:

(4) $$\begin{array}{l} H^{1}=\alpha {\left(I-\left(1-\alpha \right)\;A\right)}^{-1}H^{*} \end{array}$$

where H^*^ is the node embedding vector matrix obtained by the aforementioned convolution operation.

Since the time complexity of Equation (4) is $O\left(n^{2}\right)$, when the network scale is large, it may be expensive. Therefore, we introduce the iterative form of Equation (4):

(5) $$\begin{array}{l} Z^{0}=H^{*},\;Z^{k+1}=\left(1-\alpha \right)\;AZ^{k}+\alpha Z^{0} \end{array}$$

It is easy to prove that $\underset{K\to \infty }{\text{lim}}$ *Z*^*K*^ = α(*I* − (1 − α) *A*)^− 1^H^*^.

Because all drug node pairs have edges of chemical structure similarity, there may be two edges between some drug node pairs. The same is true for target node pairs, and will bring inconvenience to the random walk. To simplify the problem, we delete the edges representing the similarity of drug structure and protein sequence from the heterogeneous network. That is, the graph convolution operates on a complete heterogeneous network whereas the random walk is only performed on a sub-network of the complete network.

### Decoder

While encoder maps each node in the heterogeneous network to a real-valued embedding vector, the decoder reconstructs the original network from the embedding vectors. The decoder is essentially a scoring function $s\left(u,r,v\right):{\mathbb{R}}^{d}\;\times \;R\times \;{\mathbb{R}}^{d}\to \mathbb{R}$, used to score the triplets (*u, r, v*) so that we can evaluate the probability of edge *r* existing between *u* and *v*, where *u* and *v* are nodes, and *r* is a certain type of edge.

In our experiments, we use DistMult (Yang et al., 2015) as the decoder, which is known to perform well on standard link prediction benchmarks. The scoring function is:

(6) $$\begin{array}{l} s\left(u,r,v\right)={e_{u}}^{T}M_{r}e_{v} \end{array}$$

where *e*_*u*_ and *e*_*v*_ are the embedding vectors of *u and v*, respectively. ${e_{u}}^{T}$ is the transpose of *e*_*u*_, and $M_{r}\in {\mathbb{R}}^{d\times d}$ is an edge-type specific diagonal matrix.

In terms of Equation (6), we can reconstruct the original networks. Take the reconstruction of a DTI network as an example:

(7) $$\begin{array}{l} DTI_{re}={V_{drug}}^{T}M_{DTI}V_{protein} \end{array}$$

where *V*_*drug*_ and *V*_*protein*_ are the matrices of drug embedding vectors and target embedding vectors, respectively, and *M*_*DTI*_ is the diagonal matrix used to reconstruct the DTI network.

### Training

The loss of network reconstruction is as follows:

(8) $$\begin{array}{l} {ℒ}_{re}=\sum_{r\epsilon R}\sum_{i,j}{\left(Q_{ij}\right)}^{2},Q\\ \;=P(Network_{original}^{r}-Network_{reconstruction}^{r}) \end{array}$$

where $Network_{original}^{r}$ and $Network_{reconstruction}^{r}$ are the original network with edge type *r* and the corresponding reconstructed network, respectively. *P* is a mask matrix where *P*_*ij*_ = 1 indicates that the element in the *i*-th row and *j*-th column of $Network_{original}^{r}$ appears in the training set, otherwise it does not occur. *Q* is a matrix that stores the difference between the predicted value and the ground truth in the training set. We further add the regularization term of the weight coefficient to obtain the objective function:

(9) $$\begin{array}{l} {ℒ}_{re}=\sum_{r\epsilon R}\sum_{i,j}{\left(Q_{ij}\right)}^{2}+\lambda \sum_{w}w^{2},\;Q\\ \;=P(Network_{original}^{r}-Network_{reconstruction}^{r}) \end{array}$$

Our optimization goal is to minimize Equation (9), where $\underset{w}{\sum }w^{2}$ is the sum of the squares of all the weights, and λ is an adjustment coefficient. In GADTI, there are three trainable parameters: (1) four matrices for initializing node vectors, i.e., *W*^*drug*^, *W*^*disease*^, *W*^*protein*^and *W*^*sideeffect*^; (2) 12 edge-type-specific neural network weight matrices $W_{r}^{0}$ for aggregating neighborhood information; and (3) 8 edge-type-specific diagonal matrices *M*_*r*_ used to reconstruct the networks.

We adopted the same sampling strategy and dataset division strategy as Wan et al. (2019). For the DTI network, the sample pair with an edge connection is regarded as the positive sample, and the sample pair without a connection is the negative sample. We randomly collect 10 negative samples for each positive sample to form the DTI dataset used by the model. Ten-fold cross-validation (Le et al., 2019) was used for performance evaluation. In each fold, the DTI dataset is randomly divided into three independent parts: training set, validation set and test set, with ratios of 0.855, 0.045, and 0.1 respectively. The training set of GADTI is composed of the training set of DTIs and other seven datasets. In each iteration, we update the model parameters on the training set, and then evaluate the model on the validation set. If the new model parameters show better performance on the validation set than before, the test set will be used to test the generalization ability of the model.

In addition to L2 regularization, early stopping is introduced to alleviate over-fitting. If the performance of the model on the validation set does not increase for *n* iterations, it can be considered that overfitting has occurred, so the training will stop early. Adaptive moment estimation algorithm (Adam) (Kingma and Ba, 2015) is selected to minimize the objective function. The dimension of embedding vector and the learning rate are set to 1,000 and 0.001, respectively, according to independent experiments. Our code runs on PyTorch V1.7 and DGL V0.5.

## Results

### Performance Evaluation

We used 10-fold cross-validation to test the performance of our algorithm, and stratified sampling to ensure that the proportion of samples in each category in the training set and test set were the same as in the original dataset. The area under the receiver operator characteristic curve (AUROC) (Le, 2019) and the area under the precision–recall curve (AUPRC) were chosen to evaluate the performance of our approach and baseline methods.

The receiver operator characteristics (ROC) curve is suitable for evaluating the overall performance of the classifier because it takes both positive and negative samples into consideration (Le et al., 2020). However, class imbalance often occurs in actual datasets. For example, in a DTI network, the number of negative samples is much larger than that of positive samples. In this case, the ROC curve presents an overly optimistic estimate of the effect. Conversely, both indicators of the precision–recall (PR) curve focus on positive samples. In the class imbalance cases, people are mainly concerned with positive samples, and thus the PR curve is widely considered to be better than the ROC curve. We use both AUROC and AUPRC. The larger the value of AUROC and AUPRC, the better the performance of the method.

### Comparison With Baseline Methods

To evaluate the performance of GADTI, we compared it with four popular computational methods: MSCMF (Zheng et al., 2013), TL_HGBI (Wang et al., 2014), DTINet (Luo et al., 2017), and NeoDTI (Wan et al., 2019). These methods all predict DTIs from a heterogeneous network composed of multiple datasets. MSCMF uses matrix factorization methods and linear combinations of matrices to achieve prediction. TL_HGBI first establishes a three-layer heterogeneous network consisting of disease, drug, and protein data, and then uses an iterative strategy for drug repositioning. Meanwhile, DTINet focuses on learning low-dimensional vector representations of features that can accurately interpret the topological characteristics of each node in a heterogeneous network, and then makes predictions based on these representations through a vector space projection scheme. NeoDTI is close to the non-random walk version of GADTI. It first aggregates neighborhood information, and then reconstructs the network through two bilinear transformations. We run all five methods on the same dataset and implement three rounds of 10-fold cross-validation to compare their performance. The hyperparameters used in the baseline methods are the same as those in NeoDTI.

**Figure 4:**
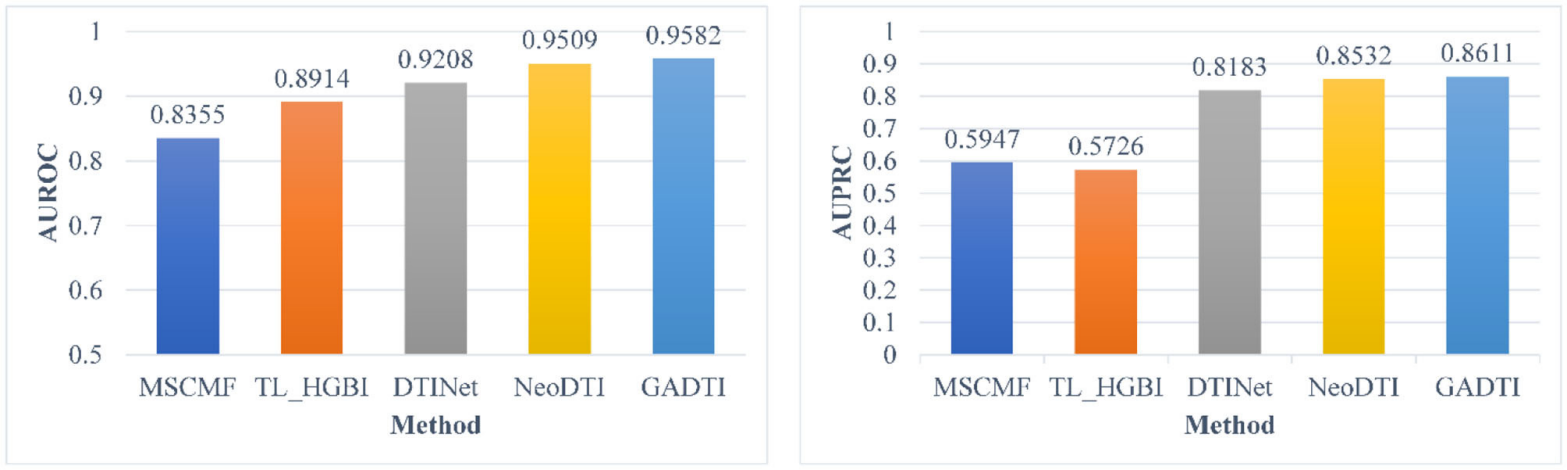
Comparison between MSCMF, TL_HGBI, DTINet, NeoDTI, and GADTI in terms of AUROC and AUPRC based on 10-fold cross-validation (#positive: #negative = 1:10).

**Figure 5:**
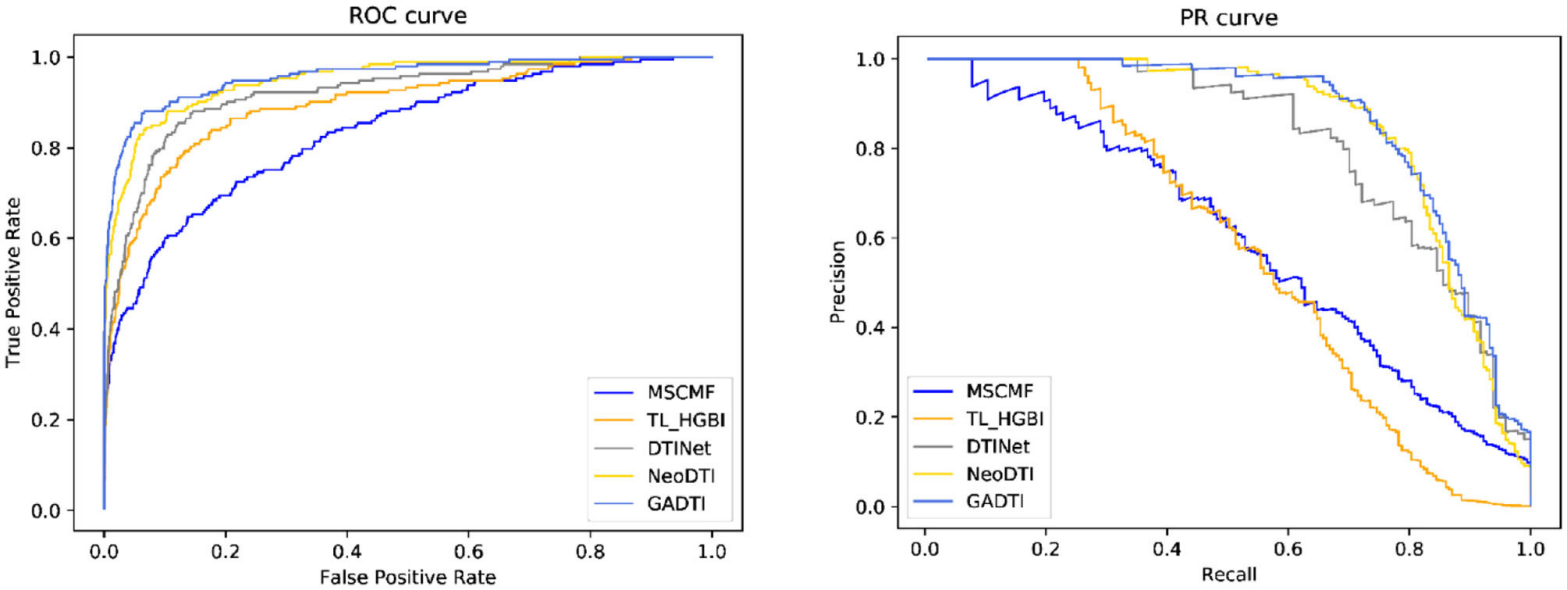
The ROC curves and PR curves of different methods (#positive: #negative = 1:10).

When the ratio of positive sample to negative sample is 1:10, the results of GADTI and the baseline methods are shown in Figures 4, 5. We observe that GADTI has an AUROC value of 0.9582, which is higher than those of NeoDTI (0.9509), DTINet (0.9208), TL_HGBI (0.8914), and MSCMF (0.8355). Meanwhile, in terms of AUPRC, which is more suitable for the current class imbalance case, GADTI is also better than all the baseline methods. Our approach slightly outperforms the second-best method (0.73% in terms of AUROC and 0.79% in terms of AUPRC).

**Figure 6:**
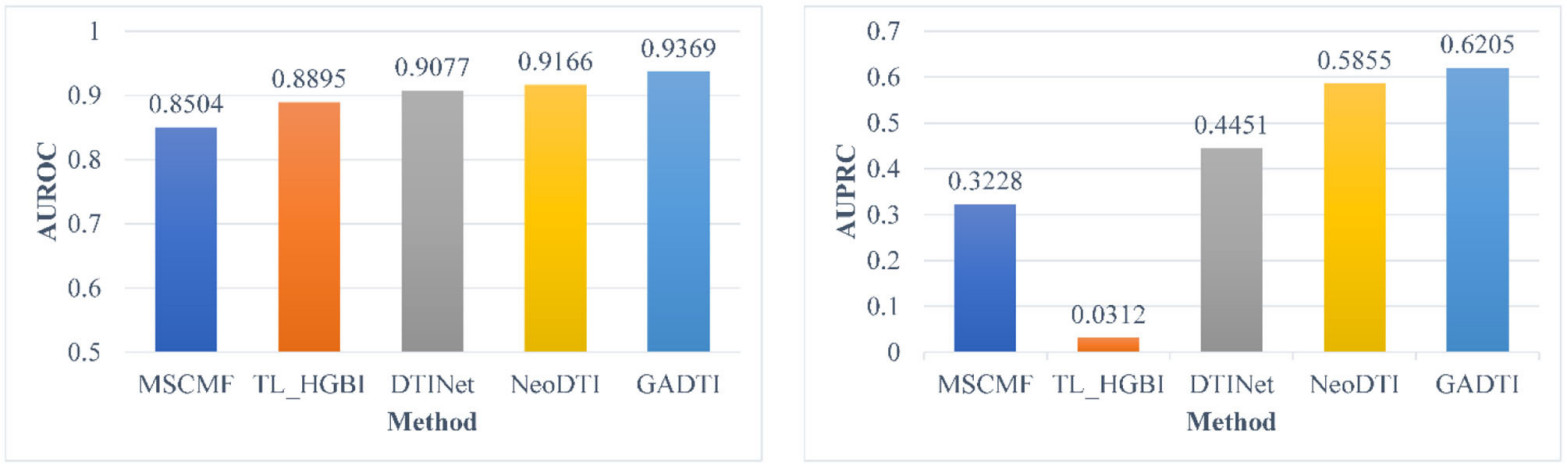
Comparison between different methods in terms of AUROC and AUPRC based on 10-fold cross-validation (all unknown pairs were treated as negative examples).

Some DTI prediction methods based on machine learning include all unknown DTIs (treated as negative examples) in the training. To have a better comparison, we did additional test in this scenario. Experiment shows that GADTI still achieve the best performance, with an AUROC of 0.9369 and an AUPRC of 0.6205, and it stays ahead by a bigger margin. We notice that the AUROC values of all methods range from 0.8504 to 0.9369, but the AUPRC values range from 0.0312 to 0.6205, which is a large gap. Figure 6 shows the experimental results of the dataset including all unknown DTIs.

**Figure 7:**
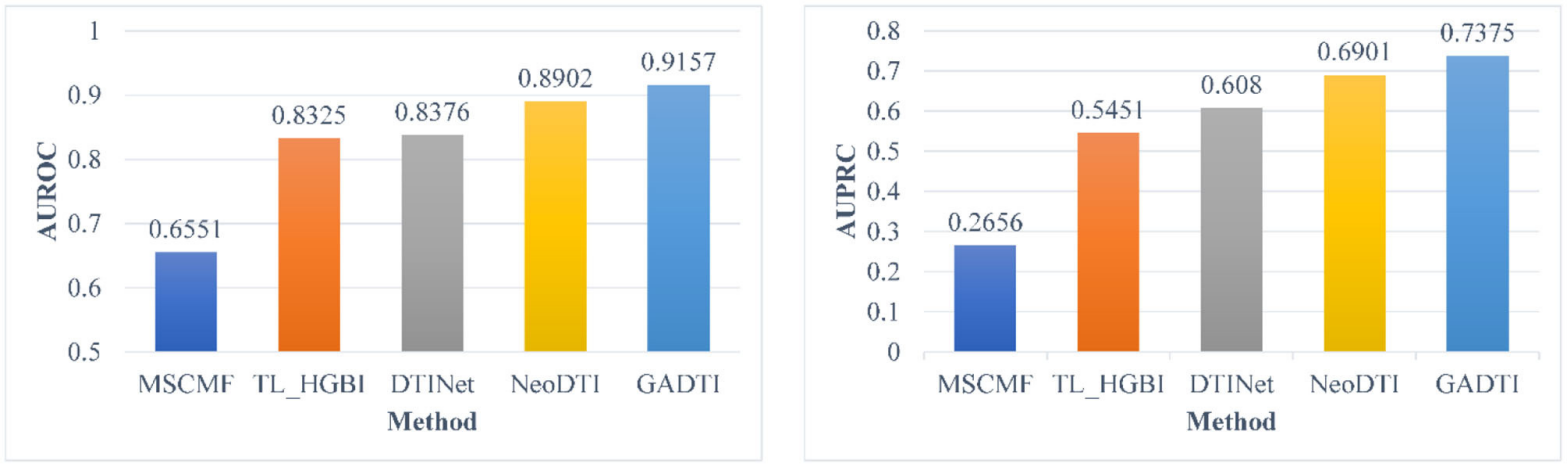
Comparison between different methods in terms of AUROC and AUPRC based on 10-fold cross-validation (#positive: #negative = 1:10, DTIs with similar drugs or targets were removed).

The dataset in section Dataset contains homologous proteins or structurally similar drugs, which reduces the difficulty of predicting their interactions. In other words, the good performance of the DTI prediction method may come from a simple algorithm rather than a well-designed algorithm. Therefore, we carried out an additional experiment which is the same as in Wan et al. (2019): the DTIs with homologous proteins (similarities > 0.4) or similar drugs (similarities > 0.6) were removed. Figure 7 shows the experimental results, where the ratio of positive samples to negative samples is 1:10. GADTI greatly outperforms the second-best method (2.55% in terms of AUROC and 4.74% in terms of AUPRC).

### Case Study

To evaluate the prediction performance, we downloaded the latest approved DTI dataset (V5.1.8, 2021-01-03) from DrugBank to verify the DTIs predicted by GADTI. We generated a set *DTI*_*newly*, which contained 1,040 new DTIs related to our original dataset from the latest DTI dataset. For each fold, top 40 potential DTIs were selected for each drug based on their predicted scores. Because the experiment used three rounds of 10-fold cross-validation, we obtained 30 tables with 708 rows and 40 columns. Each row represented the potential DTIs of a drug. The DTIs of each drug were then sorted in descending order by the number of occurrences. A predicted DTIs set *DTI*_*pre* was generated by selecting the top *m* DTIs for each drug. Finally, the number of DTIs (hit number) in the intersection of *DTI*_*newly* and *DTI*_*pre* was calculated to verify the reliability of the prediction. The results are shown in Table 2.

**Table 2:** Hit numbers of GADTI in different configurations.

	***m* = 10**	***m* = 20**	***m* = 30**	***m* = 40**
Configuration A: #positive: #negative = 1:10	211	406	508	570
Configuration B: all unknown pairs were treated as negative examples	291	402	475	523
Configuration C: #positive: #negative = 1:10, and DTIs with similar drugs or targets were removed	149	265	351	422

We observe that 54.8% of all new DTIs are predicted by GADTI in case of m = 40.

## Discussion

Finding novel DTI pairs is of great significance for drug development. However, biochemical experiments are very costly and time-consuming. Therefore, computational methods have attracted much attention recently because they can quickly and cheaply evaluate potential DTIs. Early DTI prediction studies are mainly divided into two categories: (a) inferring based on drug similarity and target similarity (Chen et al., 2012; Cheng et al., 2012; Mei et al., 2013; Wang et al., 2014; Seal et al., 2015); and (b) binary prediction based on drug feature and target feature (Nagamine et al., 2009; Lan et al., 2016; Olayan et al., 2018; Chen et al., 2019; Shi et al., 2019). The GADTI approach proposed in this paper also utilizes similarity data and the features of drugs and targets, which are represented in vectors. However, unlike previous studies, the network embedding method and the graph autoencoder framework are introduced to learn the embedding feature vectors of drugs and targets from multi-source heterogeneous networks for predicting unknown DTIs.

We use AUROC and AUPRC to evaluate the performance of GADTI and the baseline methods. The results show that GADTI greatly outperforms the other methods in three different scenarios. Only NeoDTI achieves comparable results under the situation where the ratio of positive sample to negative sample is 1:10 (Figure 4). This may be because NeoDTI also adopts GCN for aggregating and updating. In case study, GADTI accurately predicted 54.8% of the new DTIs (Table 2). We observe that the hit numbers of configuration B are less than those of configuration A, in case of *m* = 20, 30, and 40. However, the gap decreases with the decrease of *m*. We can see that in case of *m* = 10, the result is just reversed: the hit number of configuration B is much greater than that of configuration A. A reasonable inference is that configuration B, all unknown pairs are treated as negative examples, can make the ranking of potential DTIS more accurate. As a result of our experiments we conclude that, compared with baseline methods, GADTI is more reliable and effective in discovering potential DTIs. Hence, it can be used to identify new targets for existing drugs.

The reason why GADTI performs well is that it aggregates multi-hop neighborhood information and avoids over-smoothing. First of all, GADTI uses a GCN to aggregate first-order neighbor information from heterogeneous networks to update node representation. Then, an RWR is carried out on the whole network to spread the influence of nodes. The combination of the GCN and RWR introduces multi-hop information for node feature updating. It extends the scope of information aggregation from the first-order neighborhood to the higher-order neighborhood, which is equivalent to increasing the receptive field of convolution, thereby realizing long-range message passing.

Although GADTI has made outstanding achievements in DTI prediction, it still has room for improvement. For new nodes of drugs or targets that did not appear during training, GADTI cannot directly predict their interaction with known nodes, that is, it needs to restart training to make predictions. In addition, GADTI cannot predict isolated new nodes that are not associated with known drugs or target nodes. In future research, we will introduce node features and improve the model structure to try to solve these two problems.

## Data Availability Statement

Publicly available datasets were analyzed in this study. This data can be found at: https://github.com/shulijiuba/GADTI.

## Author Contributions

ZL and QC conceived the project, developed the prediction approach. ZL and WL designed and implemented the experiments. ZL, HP, XH, and SP analyzed the result. ZL wrote the paper. All authors read and approved the final manuscript.

## Conflict of Interest

The authors declare that the research was conducted in the absence of any commercial or financial relationships that could be construed as a potential conflict of interest.
